# Genetic discrimination still casts a large shadow in 2022

**DOI:** 10.1038/s41431-022-01194-8

**Published:** 2022-09-26

**Authors:** Yann Joly, Gratien Dalpe

**Affiliations:** Department of Human Genetics, Centre of Genomics and Policy, McGill University, Montréal, QC Canada

**Keywords:** Ethics, Genetic markers

Genetic discrimination (GD) is not new. It is usually understood as a type of discrimination based on genetic characteristics that is intended to infringe or has the effect of infringing on human rights, fundamental freedoms, and human dignity [1]. Like sexual, ethnic, or disability-based discrimination, GD can be a source of exclusion. It may limit a person’s social and professional opportunities and lead to psychological, social, and economic disadvantage and distress [2]. The first cases of GD surfaced in the fields of personal insurance and employment in the United States in the late 1970s. Since then, many countries in America, Europe, Asia, and Oceania have adopted laws to prevent this kind of discrimination. In low- and middle-income countries with less capacity to perform genetic tests on a broad scale and facing pressing health and economic challenges, non-GD laws are rarely a priority [3].

Given these legal developments, one may wonder why in 2022, GD remains a problem in biomedical research and precision medicine. In fact, this issue remains so pervasive that one of the first questions asked by individuals that are considering undergoing a research, or clinical genetic (or other OMICS), test is very often ‘will this impact my insurability or that of my children?’ or ‘will the results be kept completely confidential from inquiring third parties?’. If the response given to them is not sufficiently reassuring, these persons will then likely decline the opportunity to undergo the genetic test [4]. This is surely not an ideal scenario given the growing importance of genetics and its related disciplines for precision medicine. Such enduring preoccupation led to the creation, in 2018, of the Genetic Discrimination Observatory (GDO), an international consortium of researchers assembled to carry out research on GD and provide novel solutions to address this problem [5]. Since then, the GDO has identified several of the factors behind the persistence of GD, they include (1) the challenge of preventing new types of ‘OMICS’ discrimination through genetic-specific legislation, (2) the lack of empirical evidence on GD (3) insufficient information on existing prevention measures for non-expert stakeholders, (4) new stakeholders outside the traditional fields of insurance and employment are getting interested in using genetic tests, (5) a strong popular belief that GD is a risk associated with genetics regardless of evidence and, of existing protections (6) as well as, the uncertainty about which legal protection applies for international research projects [6]. To fully envision the extent of the problem, one should also consider that the large quantity of genetic data held in academic, commercial and government databases. Furthermore, because of the familial nature of genetic data, databases hold information that can be used not only to attempt to re-identify data donors but also their blood relatives. This means that even if someone has never provided a sample for genetic testing, their genetic information can likely be inferred using information from one of their relatives who did [7]. Furthermore, it is also possible to associate, with some degree of success, the presence of genetic markers of disease with specific population groups, thus opening the door for racist groups to use genetic results to further stigmatize already vulnerable populations.

Are we moving towards the genetic dystopia portrayed in the cult movie GATTACA, or the geneticization of society described in the writings of Abbey Lippman in the 1990s [8]? Do current GD risks warrant a review of the current pro-data sharing trend increasingly prevailing in the genomic community since the completion of the Human Genome Project? These are hard questions that geneticists and genomic researchers can’t afford to shy away from. While GD casts a large shadow, it is not a widespread issue and generally happens in well-delineated circumstances (ex. access to life insurance, employment in the U.S., for people having tested positive for highly predictive monogenic late-onset serious conditions), according to limited quality studies available on the topic [9]. Arguably, considering GD’s dire negative impact on a person, such cases are still too numerous. There are also worrying signs that with the rapid development of OMICS sciences, GD is becoming a more widespread. The willingness of international organizations and national legislators in many countries to position themselves and act against this type of discrimination is reassuring. Yet to be truly efficient, legislative efforts will need to shift from reactionary to more anticipatory, coordinated and evidence-based efforts.

Lately, a particular area of GD concern is that of national governments’ use of ‘OMICS’ data for biometrics and other purposes in law enforcement, immigration, and national defense. Indeed, while national governments can be relied upon—to a significant extent—to legislate to prevent the misuse of genetic information by private actors, they have much less incentive to limit their own capacity to use genetic data. In this context, national DNA forensic databases for profiling and crime investigation purposes have grown exponentially in the last 20 years (See for G7 countries in Table 1). This specific challenge led the ESHG in 2021 to issue a warning against the misuse of genetic tests and biobanks for discrimination purposes against ethnic minorities [10]. Yet, the debate is complex as some uses of genetic data for law enforcement, for example, to facilitate the identification of dangerous criminals, can contribute to important public good objectives. In this context, finding an appropriate balance between the protection of individual rights and the collective interest will be determinant. In any circumstances, the use of genetic data by government agencies for purposes conducive to oppression, stigmatization or discrimination of equity-seeking population groups should be proscribed by human rights international organizations and strongly denounced by members of the international community.

**Table 1:** Genetic Discrimination in G7 countries.

Countries	Protection measures	Legally Binding?	Sectors covered	Date adopted	Evidence of GD (excluding government use)	Government controlled DNA databases (individual profiles)
Canada	Genetic Non-Discrimination Act	Yes	General prohibition of GD in the public sector, additional protection to prevent GD in the conclusion of ‘agreements’ in the private sector	2017	Yes	National DNA Data Bank, over 622 thousand DNA profiles (March 2022 data)
France	Loi du 4 mars 2002 relative aux droits des malades	Yes	General prohibition of GD, with specific sector legislation	2002	None documented	Fichier national automatisé des empreintes génétiques, over 5,21 million DNA profiles (December 2021 data)
Germany	Human Genetic Examination Act	Yes	General prohibition of GD, with specific provisions for insurance (with some exclusions) and employment	2009	Yes, 1 small-scale study	German Forensic DNA Database, over 826 thousand DNA profiles (December 2021 data)
Italy	Personal Data Protection Code & General Authorisation for the Processing of Genetic Data (reviewed on an annual basis)	Yes	Genetic data can only be processed in the context delineated annually by the General Authorisation for the Processing of Genetic Data	2002	None documented	Banca Dati Nazionale del DNA, over 240 thousand DNA profiles (June 2020 data)
Japan	None	N/A	N/A	N/A	Yes (1 large-scale survey)	National Police Agency DNA database, over 1.29 million DNA profile (December 2019 data)
United Kingdom	Code on Genetic Testing and Insurance	No	Personal insurance (with some exceptions)	2001 (Concordat and Moratorium on Genetics and Insurance)	None documented in the past 15 years	National DNA Database (NDNAD), over 5.82 million DNA profiles (June 2022 data)
United States	Genetic Information Nondiscrimination Act	Yes	Health insurance, employment, group insurance plan (more protection maybe afforded at the state level)	2008	Yes	National DNA Index System (NDIS), over 14.83 million DNA profiles (October 2021 Data)

The GDO research so far suggests that GD could be most successfully addressed through a three-pronged approach consisting in: (1) introducing greater flexibility in our anti-GD laws to allow them to evolve at a faster pace, closer to that of genetic technology and GD risks, (2) providing accessible, up-to-date information about the risks of GD and existing protections to all stakeholders and, (3) working at the international level through the GDO to rapidly communicate emerging GD risks to national governments, build consensus on broadly defined minimal levels of GD protection for all countries and, provide information on GD to stakeholders contributing to international genetic research. For many patients suffering from life-threatening diseases, genetic research offers the only hope of a cure. Such research shouldn’t be unduly jeopardized by the threat of GD. In the end, the benefits of genetic research speak for themselves and advocate in favour of moving ahead with a responsible approach to genetic and genomic data sharing that will give due consideration to GD risk. Locking genetic data in virtual strongboxes would send the wrong message that there is something inherently sensitive and stigmatizing about this information and ossify important health research and services.
