# Correction: Genetic discrimination still casts a large shadow in 2022

**DOI:** 10.1038/s41431-022-01229-0

**Published:** 2022-11-07

**Authors:** Yann Joly, Gratien Dalpe

**Affiliations:** Department of Human Genetics, Centre of Genomics and Policy, McGill University, Montréal, QC Canada

**Keywords:** Ethics, Genetic markers

Correction to: *European Journal of Human Genetics* 10.1038/s41431-022-01194-8, published online 26 September 2022

The article “Genetic discrimination still casts a large shadow in 2022”, written by Yann Joly et al., was originally published electronically on the publisher’s internet portal on 26 September 2022 without open access. With the author’ decision to opt for Open Choice the copyright of the article changed on 30 October 2022 to © Author(s) 2022 and the article is forthwith distributed under a Creative Commons Attribution 4.0 International License, which permits use, sharing, adaptation, distribution and reproduction in any medium or format, as long as you give appropriate credit to the original author(s) and the source, provide a link to the Creative Commons licence, and indicate if changes were made. The images or other third party material in this article are included in the article’s Creative Commons licence, unless indicated otherwise in a credit line to the material. If material is not included in the article’s Creative Commons licence and your intended use is not permitted by statutory regulation or exceeds the permitted use, you will need to obtain permission directly from the copyright holder. To view a copy of this licence, visit http://creativecommons.org/licenses/by/4.0.

The original article has been corrected.

